# CircRNA circFOXK2 facilitates oncogenesis in breast cancer via IGF2BP3/miR-370 axis

**DOI:** 10.18632/aging.203347

**Published:** 2021-07-30

**Authors:** Wei Zhang, Hui Liu, Junjie Jiang, Yunyun Yang, Wenjie Wang, Zhengyan Jia

**Affiliations:** 1 Department of Thyroid and Breast I, Cangzhou Central Hospital, Cangzhou, Hebei Province, China; 2 Outpatient Comprehensive Treatment, Cangzhou Central Hospital, Cangzhou, Hebei Province, China; 3 Department of General Surgery, Botou Hospital, Botou, Hebei Province, China; 4 Department of General Surgery, Qingxian People’s Hospital, Qingxian, Hebei Province, China

**Keywords:** breast cancer, metastasis, circFOXK2, IGF2BP3, miR-370

## Abstract

Metastasis is the leading cause of breast cancer (BC)-related deaths. Circular RNAs (circRNAs) have emerged as essential regulators for cancer progression and metastasis. Therefore, the objective of this study was to investigate the role of circRNAs in BC metastasis and related mechanism. In this study, we established the BC cell line with high or low potential of metastasis. RNA sequencing, migration and invasion assay, Fluorescence *in situ* hybridization, luciferase report assay, circRNA pulldown, and transmission electron microscopy were performed to elucidate the molecular mechanism. The results showed that circRNA circFOXK2 was significantly increased in BC cells with high metastatic ability, and the upregulation of circFOXK2 was correlated with poor clinicopathological characteristics. Functional experiments demonstrated that overexpression of circFOXK2 promoted migration and invasion of BC cells. Also. circFOXK2 could act with IGF2BP3, an RNA-binding protein, and miR-370 to synergistically promote BC metastasis. Moreover, miR-370 could be transferred through exosomes to enhance the metastatic ability of recipient cells. In conclusion, circFOXK2 functions as a key regulator in BC metastasis, and the role of circFOXK2 on BC metastasis is tightly associated with the involvement of IGF2BP3 and miR-370. CircFOXK2 might serve as a potential biomarker for the diagnosis and treatment of BC.

## INTRODUCTION

The incidence of cancer and cancer-associated death has been on the rise at 3.5% in China since 2000, making cancer a significant burden on public health care [1, 2]. Among diverse cancer types, breast cancer (BC) is the most common malignancy among females in China [3]. It is estimated that 70–80% of patients with early-stage and non-metastatic BC are curable, whereas BC with advanced-stage is not curable due to current diagnostic and therapeutic strategies [4]. Over the past decades, the treatment of BC has dramatically evolved in many aspects, including surgery, radiotherapy, chemotherapy, hormonal manipulation, or a combinational treatment [5]. The survival rate of BC, however, is still low and displays heterogeneous patterns in different regions thanks to the lack of early diagnosis and cost-effectiveness of treatments [6].

It has been demonstrated that the majority of BC-related deaths result from metastasis to other organs rather than the primary tumor itself [7]. To date, identification of BC metastasis is to detect the clinical manifestations of the metastatic organs, biopsies of metastatic organs, radiological assessments, medical imaging, as well as molecular markers [8]. Although these screenings have dramatically lowered the BC metastasis-associated mortality, these strategies mentioned above are still not sufficient and accurate for diagnosing BC at the earliest stage [7]. Therefore, it is an urgent need to explore the molecular mechanism underlying BC metastasis. Growing evidence demonstrated that metastasis is a complicated multi-step process, including invasion, intravasation, survival in blood, extravasation, as well as colonization at the distant metastatic organs [9]. As the first and most critical step of metastasis, the invasion is regarded as a migratory process of cancer cells from the primary tumor to distant other organs [10]. However, the initiation of invasion during BC progression is not fully understood.

Over the past decades, RNAs, particularly the non-coding RNAs (ncRNAs), have drawn increasing attention from both clinical and academic researchers due to the rapid development of high-throughput RNA sequencing techniques and bioinformatics [11]. Most RNAs in eukaryotic cells are ncRNAs, not messenger RNAs (mRNAs) [12]. Accumulating evidence revealed that ncRNAs, including long non-coding RNAs (lncRNAs), microRNAs (miRNAs), and circular RNAs (circRNAs), play an essential role in physiological and pathological processes, including cancers [13]. Among these ncRNAs, circRNAs have been reported to interact with miRNAs or RNA-binding proteins (RBPs) to regulate the development and progression of cancers [14, 15]. Also, circRNAs are considered as promising biomarkers for early diagnosis of cancers, such as serum cricRNAs [16]. In BC, several circRNAs are found to be aberrantly expressed in BC and participate in the carcinogenesis of BC [17–19]. However, the function of circRNAs in BC metastasis has not been comprehensively investigated.

In this study, by establishing BC cells with high or low potential of metastasis, we aimed to investigate the functional cricRNA associated with BC metastasis and related molecular mechanism. This study provides insight into the molecular basis of BC metastasis and the role of circRNA in the metastatic process.

## RESULTS

### CircFOXK2 is upregulated in highly metastatic BC cells

**Figure 1:**
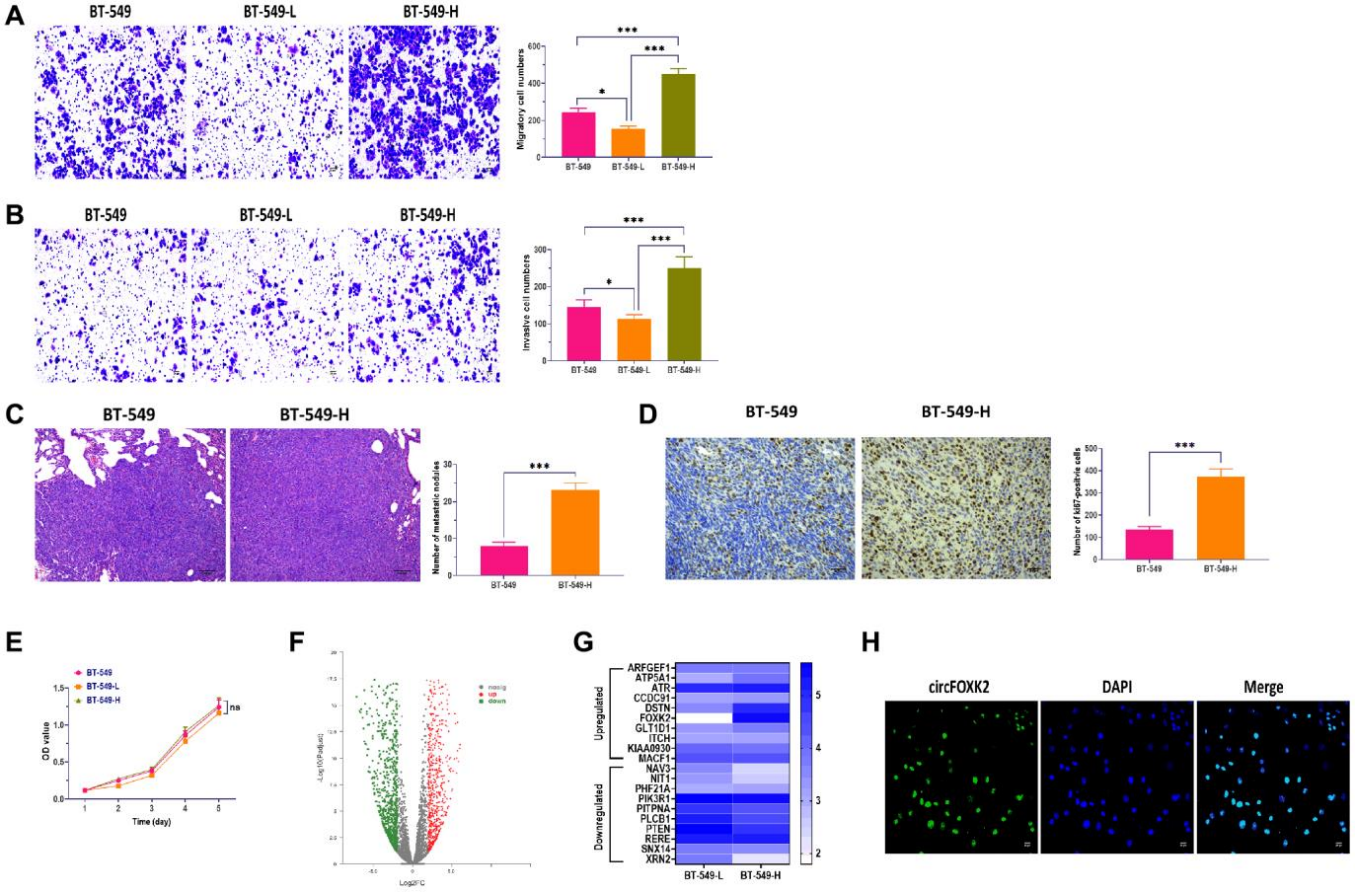
**CircFOXK2 is upregulated in highly metastatic BC cells.** (**A**) Migration ability of BT-549 cells with high and low potential of metastasis (BT-549-H and BT-549-L). Scale bar: 20 μm. (**B**) Invasion ability of BT-549 cells with high and low potential of metastasis (BT-549-H and BT-549-L). Scale bar: 20 μm. (**C**) Lung metastasis of mice injected with BT-549 or BT-549-H cells through the tail vein, as determined by H&E stain. Scale bar: 100 μm. (**D**) The expression of Ki67 in lung tissues of mice injected with BT-549 or BT-549-H cells through the tail vein, as determined by IHC assay. Scale bar: 40 μm. (**E**) Cell viability of BT-549-H and BT-549-L cells. (**F**) Volcano plot of differentially expressed circRNAs between BT-549-H and BT-549-L cells. (**G**) Heatmap for expressions of top 10 upregulated and downregulated circRNAs between BT-549-H and BT-549-L cells. (**H**) Cellular distribution of circFOXK2 in BT-549 cells, as detected by RNA FISH assay. Data were represented as mean ± SD. Each experimental group had at least three replicates. ^*^p < 0.05, ^**^p < 0.01, ^***^p < 0.001.

To investigate the mechanism by which BC cells are highly metastatic, we established two BT-549 cell populations with high and low potential of metastasis (BT-549-H and BT-549-L), as previously described [20, 21]. Then, we determined the characteristics of BT-549-H and BT-549-L cells. The results showed that BT-549-H cells had a higher level of migratory and invasive abilities compared with BT-549-L and BT-549 cells (Figure 1A and 1B). Also, injecting through the tail vein of mice, we found more lung metastasis and higher expression of Ki67 in mice injected with BT-549-H cells relative to BT-549-L (Figure 1C and 1D). As showed in Figure 1E, both cell populations showed similar cell viability. Next, RNA sequencing analysis was carried out to determine the differentially expressed circRNAs associated with metastasis of BC cells, as showed in a volcano plot (Figure 1F). By performing qRT-PCR, we measured the expressions of the top 10 upregulated and downregulated circRNAs, respectively. The results showed that circFOXK2 displayed the highest upregulation in BT-549-H, compared with BT-549-L (Figure 1G). Though analysis in database circBase [22], CircAtlas [23], and CircFunBase [24], circFOXK2, also named hsa_circ_0000816, was 343 nt in length and located in FOXK2 2–3 exons. Moreover, circFOXK2 was primarily expressed in the nuclei of BT-549 cells, as determined by RNA fluorescence *in situ* hybridization (FISH) assay (Figure 1H).

### CircFOXK2 is involved in metastasis of BC

**Figure 2:**
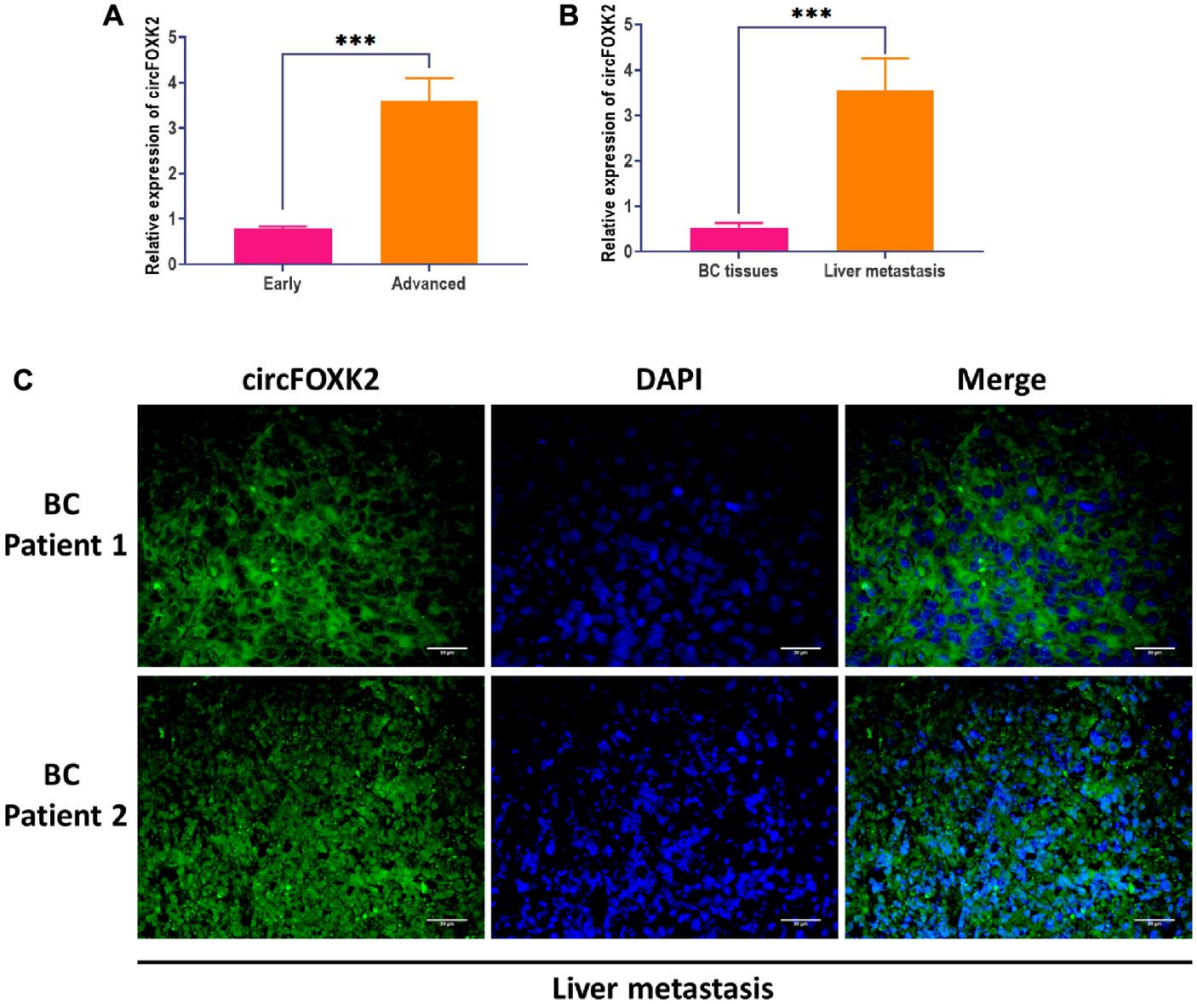
**CircFOXK2 is involved in the metastasis of BC.** (**A**) Expression of circFOXK2 in BC tissues in the early or advanced stage. (**B**) Expression of circFOXK2 in BC tissues or paired liver metastasis. (**C**) Expression of circFOXK2 in liver metastasis, as detected by RNA FISH assay. Scale bar: 30 μm. Data were represented as mean ± SD. Each experimental group had at least three replicates. ^*^p < 0.05, ^**^p < 0.01, ^***^p < 0.001.

To explore the role of circFOXK2 in metastasis of BC, we measured the expression of circFOXK2 in BC tissues and found that the level of circFOXK2 was significantly higher in advanced-stage tissues compared with early-stage (Figure 2A). Correlation analysis suggested that the upregulation of circFOXK2 was correlated with invasive histological type, lymph node metastasis, and advanced stage. For liver metastasis, the expression of circFOXK2 was higher in metastatic liver tissues compared with paired BC tissues (Figure 2B), which was verified through FISH assay (Figure 2C). These results together indicated that circFOXK2 is associated with metastasis of BC.

### CircFOXK2 is required for BC metastasis

**Figure 3:**
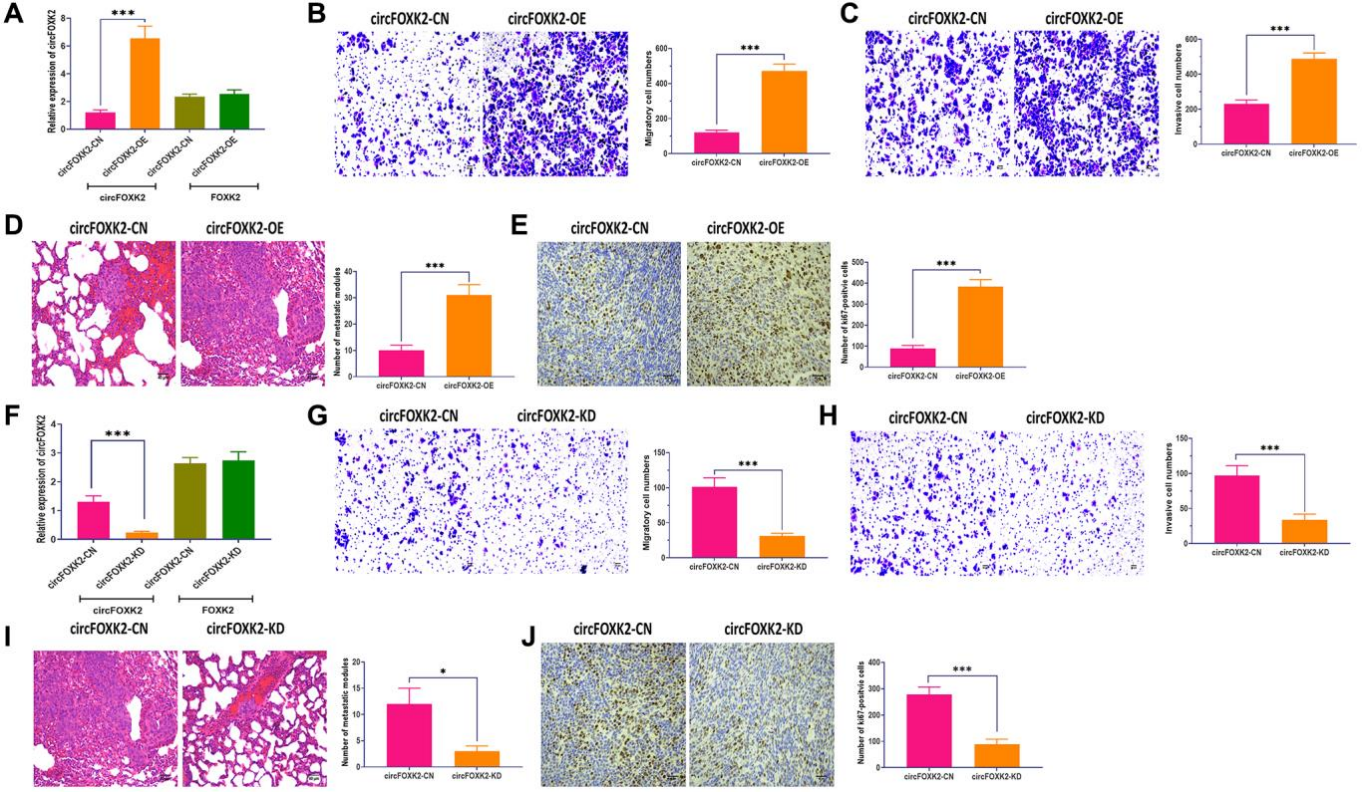
**CircFOXK2 is required for BC metastasis.** (**A**) Expressions of circFOXK2 and FOXK2 mRNA in BT-549 cells transfected with circFOXK2-expressing plasmids and the negative control. (**B**) Migration ability of circFOXK2-overexpressing BT-549 cells. Scale bar: 20 μm. (**C**) Invasion ability of circFOXK2-overexpressing BT-549 cells. Scale bar: 20 μm. (**D**) Lung metastasis of mice injected with circFOXK2-overexpressing BT-549 cells through the tail vein, as determined by H&E stain. Scale bar: 50 μm. (**E**) The expression of Ki67 in lung tissues of mice injected with circFOXK2-overexpressing BT-549 cells through the tail vein, as determined by IHC assay. Scale bar: 40 μm. (**F**) Expressions of circFOXK2 and FOXK2 mRNA in BT-549 cells transfected with circLONP2-specific anti-sense oligonucleotide (ASO). (**G**) Migration ability of circFOXK2-Knockdown BT-549 cells. Scale bar: 20 μm. (**H**) Invasion ability of circFOXK2-Knockdown BT-549 cells. Scale bar: 20 μm. (**I**) Lung metastasis of mice injected with circFOXK2-Knockdown BT-549 cells through the tail vein, as determined by H&E stain. Scale bar: 50 μm. (**J**) The expression of Ki67 in lung tissues of mice injected with circFOXK2-Knockdown BT-549 cells through the tail vein, as determined by IHC assay. Scale bar: 40 μm. Data were represented as mean ± SD. Each experimental group had at least three replicates. ^*^p < 0.05, ^**^p < 0.01, ^***^p < 0.001.

To further explore the effect of circFOXK2 on BC metastasis, we applied the plasmid of circLONP2 and circFOXK2-specific anti-sense oligonucleotide (ASO) to overexpress and knockdown the expression of circFOXK2, respectively (Figure 3A and 3F). Meanwhile, the overexpress and knockdown circFOXK2 did not influence the expression of FOXK2 mRNA (Figure 3A and 3F). In migration and invasion assays, BT-549 cells with overexpression of circFOXK2 displayed significantly increased migratory and invasive abilities compared with control cells (Figure 3B and 3C). Also, the upregulation of circFOXK2 led to a higher level of lung metastasis and Ki67 in the mouse model (Figure 3D and 3E). On the other hand, the downregulation of circFOXK2 displayed the opposite roles (Figure 3G–3J). Therefore, the results collectively demonstrated that circFOXK2 plays an essential role in BC metastasis.

### IGF2BP3 is critical for the effect of circFOXK2 on BC metastasis

**Figure 4:**
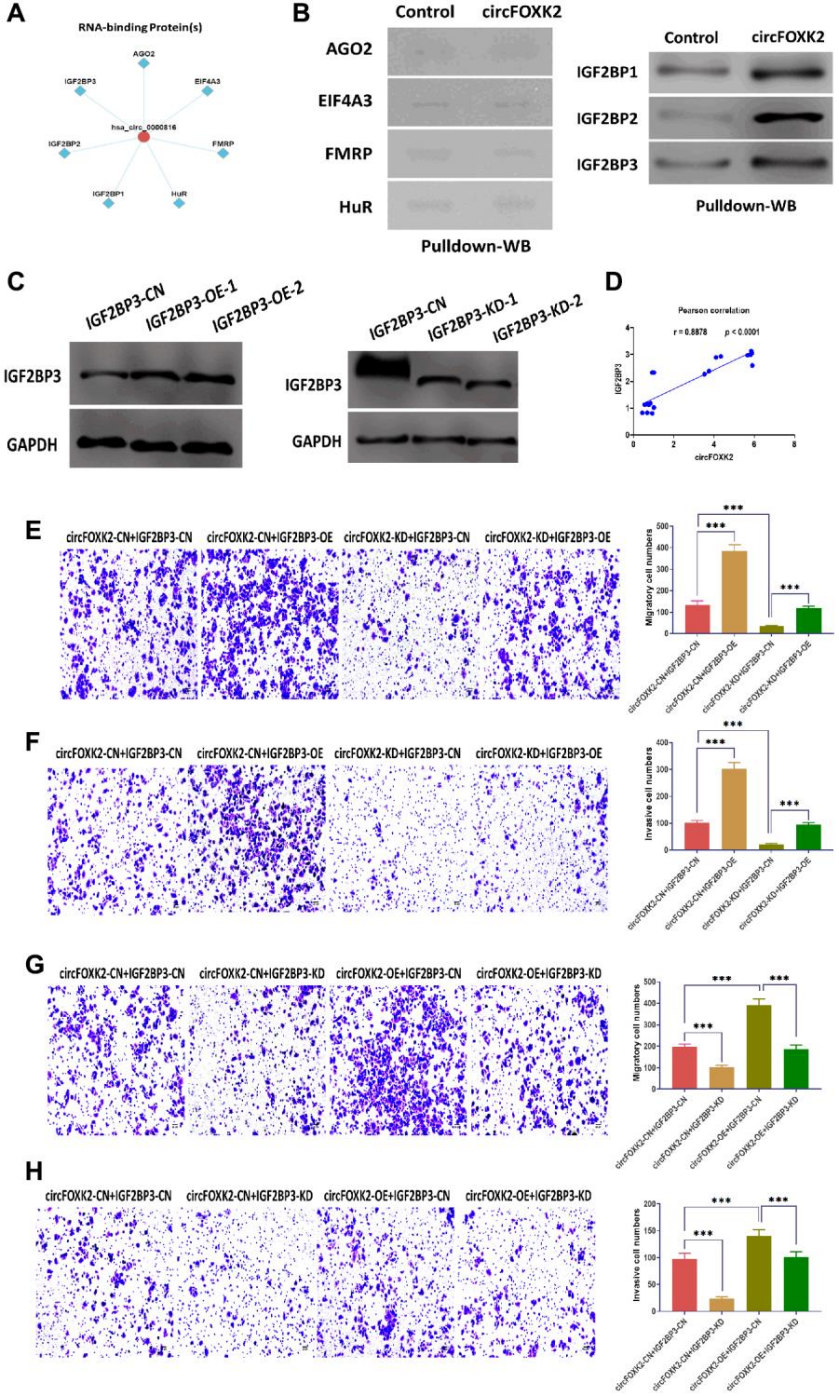
**IGF2BP3 is critical for the effect of circFOXK2 on BC metastasis.** (**A**) Prediction of RNA-binding proteins of circFOXK2. (**B**) Interaction between circFOXK2 and IGF2BP1, IGF2BP2, IGF2BP3, EIF4A3, FMRP, HuR, and AGO2, as determined by RNA pulldown and Western blotting assay. (**C**) Protein expression of IGF2BP3 in BT-549 cells transfected with IGF2BP3-expressing plasmids or IGF2BP3-specific small interfering RNA (siRNA). (**D**) Pearson correlation between the expressions of circFOXK2 and IGF2BP3. (**E** and **G**) Rescue experiments for the migration ability of BT-549 cells treated as indicated. Scale bar: 20 μm. (**F** and **H**) Rescue experiments for the invasion ability of BT-549 cells treated as indicated. Scale bar: 20 μm. Data were represented as mean ± SD. Each experimental group had at least three replicates. ^*^p < 0.05, ^**^p < 0.01, ^***^p < 0.001.

It has been demonstrated that RNA-binding proteins (RBPs) are critical for the function of circRNAs [25]. According to predictions of CircFunBase [24], several potential RBPs might be involved in the role of circFOXK2, including EIF4A3, FMRP, HuR, AGO2, and IGF2BP1-3 (Figure 4A). Of which, IGF2BP family has been reported to be essential to cancer development and progression [26–28]. By using RNA pulldown and Western blotting assays, the results showed that circFOXK2 physically interacted with three IGF2BP family members, including IGF2BP1, IGF2BP2, and IGF2BP3, but not with EIF4A3, FMRP, HuR, and AGO2 (Figure 4B). Among three, IGF2BP3 acts as a critical factor in the regulation of cancers, such as tumor cell proliferation, invasion, and chemoresistance [29]. Thus, we speculated that IGF2BP3 might be an essential RBP mediating the effect of circFOXK2 in BC metastasis. In our rescue experiments, IGF2BP3 was overexpressed and inhibited successfully in BT-549 cells (Figure 4C). The expression of circFOXK2 positively correlated with the protein level of IGF2BP3 (Figure 4D). Also, the overexpression of IGF2BP3 promoted cell migration and invasion and reversed the effect of knockdown of circFOXK2 in BT-549 cells (Figure 4E and 4F). Meanwhile, the downregulation of IGF2BP3 attenuated the effect of overexpression of circFOXK2 on migration and invasion of BC cells (Figure 4G and 4H). These results showed that IGF2BP3 is essential for the effect of circFOXK2 on BC metastasis.

### CircFOXK2-miR-370 interaction is essential for BC metastasis

**Figure 5:**
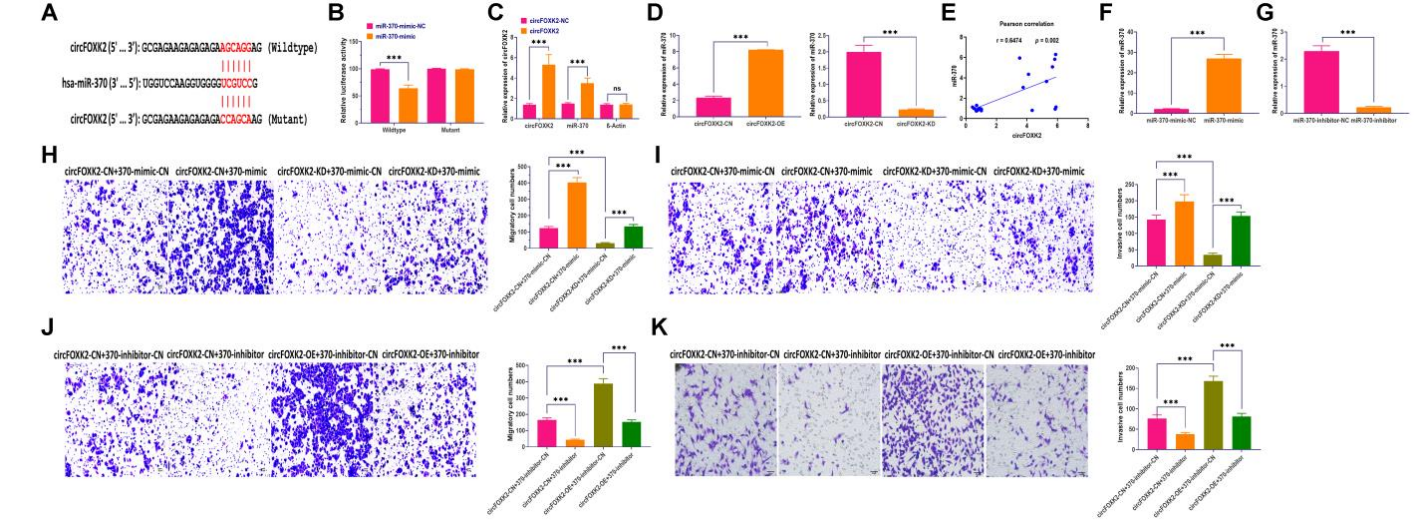
**CircFOXK2-miR-370 interaction is essential for BC metastasis.** (**A**) Putative binding site of miR-370 in the circFOXK2 sequence. (**B**) Luciferase activity in BT-549 cells treated as indicated. (**C**) circRIP assay. (**D**) Expression of miR-370 in circFOXK2-overexpressing/knockdown BT-549 cells. (**E**) Pearson correlation between the expressions of circFOXK2 and miR-370. (**F**) Expression of miR-370 in BT-549 cells treated with miR-370 mimic. (**G**) Expression of miR-370 in BT-549 cells treated with miR-370 inhibitor. (**H** and **J**) Rescue experiments for the migration ability of BT-549 cells treated as indicated. Scale bar: 20 μm. (**I** and **K**) Rescue experiments for the invasion ability of BT-549 cells treated as indicated. Scale bar: 20 μm. Data were represented as mean ± SD. Each experimental group had at least three replicates. ^*^p < 0.05, ^**^p < 0.01, ^***^p < 0.001.

The role of circRNA-miRNAs interaction has been well studied in various cancers [30, 31]. In this study, we used Circular RNA Interactome Database to predict the potential miRNAs interacting with circFOXK2 [32]. The results showed that circFOXK2 had a binding sequence of miR-370 (Figure 5A). Then, luciferase assay revealed that cells transfected with miR-370 mimic plus plasmids carrying wildtype binding sequence showed significantly decreased luciferase activity than those treated with plasmids carrying mutant binding sequence (Figure 5B). Also, circRIP assay demonstrated that miR-370 was enriched in circFOXK2-specific probes compared with those in the control group (Figure 5C). Moreover, the overexpression and knockdown of circFOXK2 increased and decreased the expression of miR-370, respectively (Figure 5D). Also, the expression of circFOXK2 displayed a positive correlation with miR-370 (Figure 5E). Collectively, these results further verified the interaction between circFOXK2 and miR-370. To further determine the role of miR-370 in BC metastasis, we applied miRNA mimic and inhibitor to overexpress and knockdown miR-370, respectively (Figure 5F and 5G). As showed in rescue experiments, the overexpression of miR-370 restored the inhibited migratory and invasive abilities in BT-549 cells with downregulation of circFOXK2, whereas the knockdown of miR-370 exerted opposite roles (Figure 5H–5K). As such, these results indicated that circFOXK2-miR-370 interaction is essential for BC metastasis.

### Exosomal miR-370 derived from BT-549-H promote BC metastasis

**Figure 6:**
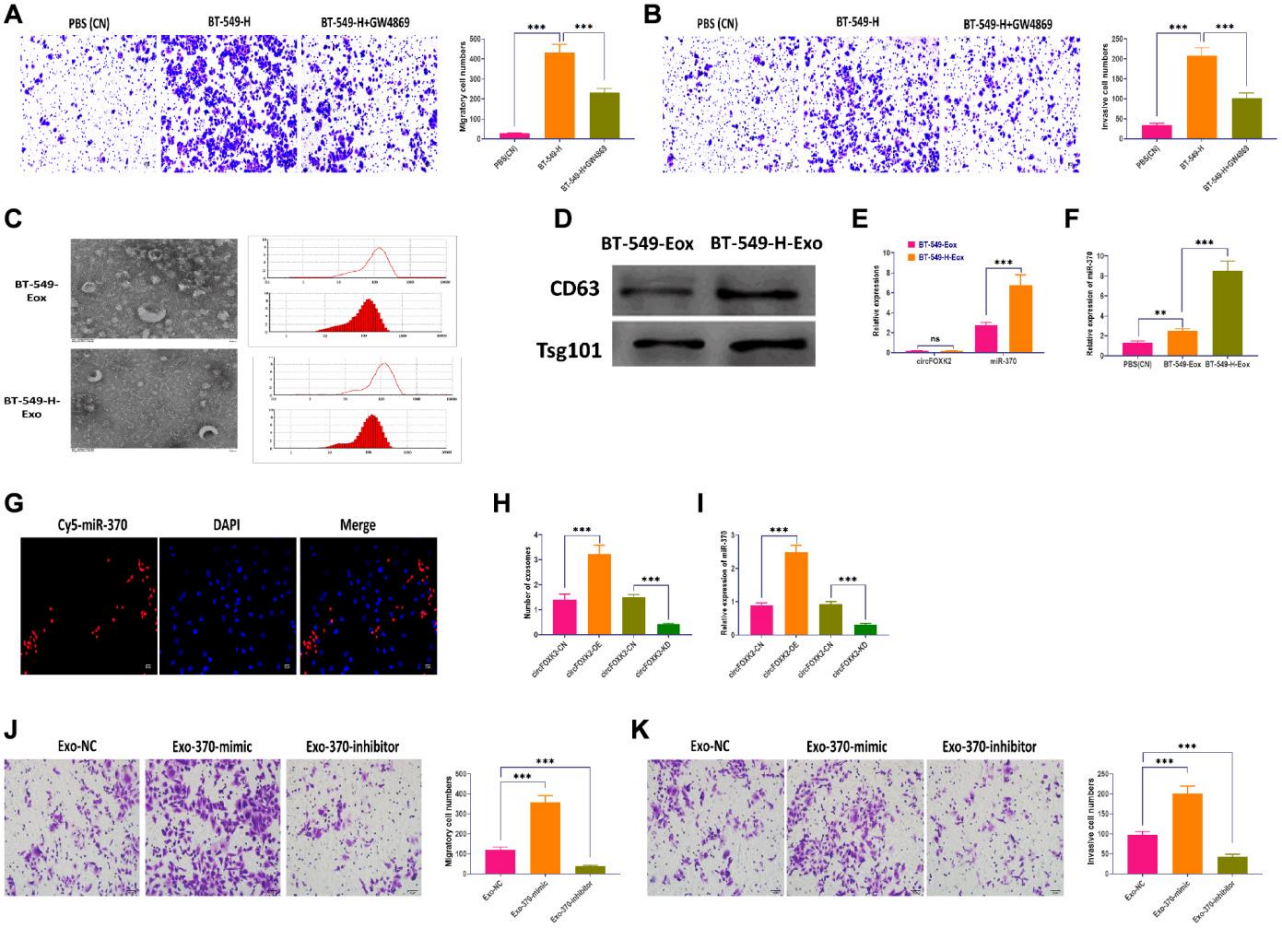
**Exosomal miR-370 derived from BT-549-H promote BC metastasis.** (**A**) Migration ability of BT-549 cells cocultured with BT-549-H cells or BT-549-H plus GW4869 treatment. Scale bar: 20 μm. (**B**) Invasion ability of BT-549 cells cocultured with BT-549-H cells or BT-549-H plus GW4869 treatment. Scale bar: 20 μm. (**C**) Morphology and size distribution of exosomes derived from BT-549 (BT-549-Exo) or BT-549-H (BT-549-H-Exo) cells, as determined by transmission electron microscopy, nanoparticle tracking analysis. (**D**) Expressions of exosomal markers CD63 and Tsg101, as detected by Western blotting. (**E**) Expressions of circFOXK2 and miR-370 in BT-549-Exo and BT-549-H-Exo. (**F**) Expression of miR-370 in BT-549 cells treated with BT-549-Exo and BT-549-H-Exo. (**G**) Exosomal miR-370 was taken up by recipient BT-549 cells, as determined by fluorescence staining assay. Scale bar: 20 μm. (**H**) Number of exosomes in BT-549 cells with overexpression or knockdown of circFOXK2. (**I**) Expression of exosomal miR-370 in BT-549 cells with overexpression or knockdown of circFOXK2. (**J**) Migration ability of BT-549 cells treated with exosomes containing miR-370 mimic or inhibitor. Scale bar: 20 μm. (**K**) Invasion ability of BT-549 cells treated with exosomes containing miR-370 mimic or inhibitor. Scale bar: 20 μm. Data were represented as mean ± SD. Each experimental group had at least three replicates. ^*^p < 0.05, ^**^p < 0.01, ^***^p < 0.001.

Exosomes play a critical role in intercellular communication in cancers; in particular, cancer cells with high metastatic potential can impact neighboring cells through exosomes [33, 34]. As shown in Figure 6A, BT-549 cells cocultured with BT-549-H displayed significantly increased migratory and invasive abilities, which was reversed the blockade of exosome generation by GW4869 [35] (Figure 6A and 6B). Then, we isolated exosomes from BT-549 (BT-549-Exo) and BT-549-H (BT-549-H-Exo) cells, respectively. The morphology and size of exosomes were identified through transmission electron microscopy, nanoparticle tracking analysis (Figure 6C). Exosomal markers CD63 and Tsg101 were measured by Western blotting assay (Figure 6D). Moreover, we found that circFOXK2 rarely expressed in exosomes, whereas miR-370 was highly expressed in BT-549-H-Exo, compared with BT-549-Exo (Figure 6E). Meanwhile, the level of miR-370 was higher in BT-549-H-Exo than BT-549-Exo (Figure 6F). Fluorescence staining assay demonstrated that exosomal miR-370 derived from BT-549-H-Exo was taken up by recipient BT-549 cells (Figure 6G). Furthermore, the overexpression and knockdown of circFOXK2 could increase and decrease both exosome generation and expression of exosomal miR-370, respectively (Figure 6H and 6I). Intriguingly, exosomes with overexpression of miR-370 promoted migration and invasion abilities in BT-549 cells, while the opposite effect was found in BT-549 cells treated with exosomes with knockdown of miR-370 (Figure 6J and 6K). These results together suggested that circFOXK2 induces metastasis of recipient cells by promoting the transfer of exosomal miR-370.

## DISCUSSION

BC initiates as a local disease and then metastasize to the lymph nodes and other distant organs [7]. Previous studies demonstrated that metastasis is the leading cause of BC-associated death [36]. Based on the widely recognized model of metastasis, a population of cancer cells in the primary tumor acquire genetic modifications over time, allowing these cells are capable of metastasizing and forming a new tumor in other distant organs [37]. Given this model, exploring the detailed mechanism of initiating cancer cell metastasizing would significantly extend our understanding of metastasis. In this study, we established a BC BT-549 cell line with a high potential of metastasis (BT-549-H) and then screened the potential circRNAs involved in the acquired metastatic ability of BT-549 cells. Also, we found that circFOXK2 was increased in BT-549-H cells and correlated with clinicopathological features of patients with BC. Moreover, the effect of circFOXK2 on BC metastasis was mediated through RNA-binding protein IGF2BP3 and miR-370. Meanwhile, miR-370 could be transferred from BT-549-H cells to recipient cells through exosomes, promoting the invasive ability of recipient cells. These results together elucidate the role of circFOXK2 in BC metastasis and related functional pathways.

In this study, by establishing a BT-549 cell population with a high potential of metastasis and RNA sequencing, we demonstrated that circFOXK2, mainly expressed in the nuclei, was associated with enhanced metastatic ability of BT-549 cell. Also, functional experiments showed that the overexpression of circFOXK2 significantly improved the migratory and invasive abilities of BT-549 cells, whereas the knockdown of circFOXK2 exerted the opposite role. According to analysis in the databases [22–24], circFOXK2, also named hsa_circ_0000816, was located in 2–3 exons of the FOXK2 gene. Qiao et al. reported that the upregulation of circFOXK2 is associated with periventricular white matter damage (PWMD) of premature infants [38]. Also, circFOXK2 may participate in the regulation of myotonic dystrophy [39]. Moreover, circFOXK2, a sponge of miR-206, is predicted to be involved in asthenospermia, a common cause of human male infertility [40]. In cancers, the role of circRNAs in metastasis has been widely recognized. For example, circ_0067934 is upregulated in both tissues and cells of hepatocellular carcinoma and promotes metastasis through miR-1324/FZD5/Wnt/β-catenin pathway [41]. Also, the downregulation of circ_100395 was associated with enhanced metastasis and poor prognosis in lung cancer [42]. Wong et al. demonstrated that circFOXK2 promotes progression and metastasis of pancreatic cancer through binding with miR-942 and RBPs complex [43]. In this study, we first that circFOXK2 is a crucial regulator for the regulation of metastasis, which provides an avenue to explore the mechanism mediating the effect of circFOXK2 on BC metastasis.

Given the multiple mechanisms underlying the function of circRNAs, the interaction between circRNAs and RBPs play a critical role in transcriptional modulation, translation, and extracellular transportation [44]. In general, RBPs are a group of proteins associated with the regulation of gene expression at either the transcriptional or translational level [25]. In this study, we demonstrated that circFOXK2 could interact with IGF2BP family members, including IGF2BP1, IGF2BP2, and IGF2BP3, in BC cells. of which IGF2BP3 and circFOXK2 synergistically regulate the metastatic ability of BC cells. Accumulating studies have been shown that IGF2BP3 functions as an essential regulator in BC progression. For example, the aberrant level of IGF2BP3 is detected in the majority of invasive triple-negative breast carcinomas [45, 46], while the expression of IGF2BP3 is only elevated in adenoid cystic carcinomas in basal-like BC [47, 48]. Furthermore, a tight correlation between IGF2BP3 expression and lymph node metastasis is found in colorectal adenocarcinoma [49], oral squamous cell carcinoma [50], and gastric cancer [51]. Collectively, IGF2BP3 functions as a fine-tuner regulating the expression of genes related to cancer progression and metastasis.

In the present study, we applied online tools to predict potential miRNA interacting with circFOXK2 and identified that circFOXK2 could directly target miR-370. As an oncogenic factor, miR-370 functions as a promoter for cancer progression through targeting TGFβ-RII [52] or FOXO1 [53, 54]. Unlike the well-studied relation that circRNAs act as miRNA sponges, we observed that the expression of circFOXK2 and miR-370 displayed a similar pattern. Functionally, our rescue experiments showed that the miR-370 and circFOXK2 synergistically regulated BC cell invasion and migration. These results indicate that circFOXK2 might interact with miR-370, thereby promoting the biogenesis of miR-370. It has been reported that circRNA ciRS-7 participates in the biogenesis of mature miR-7 [14, 55]. On the other hand, the expression of miR-370 was found to be increased in exosomes derived from BT-549-H cells, and exosomal miR-370 could be internalized by recipient cells, eventually promote invasion and migration. Collectively, our observations suggested that circFOXK2 works with miR-370 to promote BC metastasis cooperatively. However, the detailed mechanism by which how circFOXK2 interacts with miR-370 should be investigated in future studies.

In conclusion, the results suggested that circFOXK2 was upregulated in metastatic BC cells and is correlated to poor clinicopathological features of BC patients. Functionally, circFOXK2 promotes invasion and migration of BC cells, and the effect of circFOXK2 on BC metastasis is associated with the involvement of IGF2BP3 and miR-370. This study indicates that circFOXK2 might serve as a biomarker for the diagnosis and treatment of BC.

## MATERIALS AND METHODS

### Ethics statement

All patients were informed before inclusion, and the written consents were given. All experiments were approved by the ethics committee of our hospital. All animal experiments complied with the guidelines of the Animal Ethics Committee for the care and use of our hospital.

### Patients

BC primary tissues were collected from patients with BC who underwent operation between March 2015 and March 2017 at our hospital. The exclusion criteria were as follows: 1) suffering from other malignancies; 2) patients had previous treatment; 3) histologic diagnosis was not BC, and 4) patients had not complete data of analysis. All samples were stored at –80°C until use.

### Cell culture

BC cell line BT-549 and HEK293T cells were purchased from the cell bank of the Chinese Academy of Sciences (Shanghai, China). BT-549 cells were cultured in RPMI 1640 media supplemented with 10% fetal bovine serum (FBS) (Gibco, USA). HEK293T cells were cultured in Dulbecco’s modified Eagle’s medium (DMEM) with 10% FBS (Gibco, USA). Cells were maintained in an incubator (Thermo Fisher Scientific, USA) at 37°C with 5% CO_2._

### Migration and invasion assay

To establish BT-549 cell population with high and low potential of metastasis (BT-549-H and BT-549-L), BT-549 cells were subjected to repetitive invasion assay, as previously described [20, 21]. After incubation for 36 hours at 37°C invaded cells underneath the membrane and uninvaded cells were collected and expanded for the next round of screen. After 10 and 30 screen rounds, cell populations were classified as BT-549-L and BT-549-H cells, respectively. Migratory and invasive abilities were determined using QCM Chemotaxis Cell Migration Assay (24-well, 8 μm) and QCM ECMatrix Cell Invasion Assay (24-well, 8 μm) (Sigma-Aldrich, USA) according to the manufacturer’s instruction. The data were quantified in 5 random places under a microscope.

### CCK-8 assay

Cell viability was determined using Cell Counting Kit 8 (WST-8/CCK8) (Abcam, Japan) according to the manufacturer’s instructions. The BT-549-H and BT-549-L cells were seeded (1 × 10^5^ cells/well) in a 96-well dish. OD values were measured by absorbance at 460 nm at 1, 2, 3, 4, and 5 day.

### RNA sequencing

RNA sequencing assay was carried out between BT-549-H and BT-549-L cells, as previously described [56]. In brief, total RNAs were isolated from cells, exosomes, tissues using Trizol (Invitrogen, USA). RNA quality and concentration were determined by NanoDrop™ 2000 (Thermo Scientific, USA). RNA sequencing libraries were established and sequenced by Beyotime Biotechnology (Shanghai, China). Ribosomal RNAs (rRNAs) were removed from total RNAs (5 μg) using Ribo-Zero Plus rRNA Depletion Kit (Illumina, USA). Linear RNAs were digested using RNase R (New England Biolabs Inc, USA). Sequencing libraries were established using NEBNext Ultra RNA Library Prep Kit for Illumina (New England Biolabs Inc, USA) according to the manufacturer’s instructions. RNA samples were fragmented into pieces of ~ 300 bp in length, and the first-strand cDNAs were synthesized by reverse transcription and random hexamer primers. Afterward, the second-strand cDNAs were synthesized using Second Strand Synthesis Reaction Buffer. The final cDNA fragments were applied to the end modification processes, such as the addition of a single “A” base and the ligation of the adapters. Then, the chain specific libraries were constructed using USER Enzyme (New England Biolabs Inc, USA) and amplified by PCR. The libraries were qualified by NEBNext^®^ Library Quant Kit for Illumina (New England Biolabs Inc, USA). Lastly, the libraries were subjected to sequencing assay on an Illumina HiSeq sequencer system (Illumina, USA). The sequencing quality of raw data was evaluated by FastQC software [56]. High-quality reads were aligned to the human reference genome (GRCh38/hg38) using Tophat2 software [57] with default parameters. Unaligned reads were used for subsequent circRNA analysis using CIRCexplorer2 [58] and Find_circ [59]. Differential expression analysis between BT-549-H and BT-549-L was carried out using Limma (v3.32.10) R package [60]. CricRNA with *Q* value > 0.01 was defined as significantly differential expression.

### Quantitative real-time PCR

Total RNAs were isolated from cells, exosomes, tissues using Trizol (Invitrogen, USA). RNA quality and concentration were determined by NanoDrop™ 2000 (Thermo Scientific, USA). cDNA was synthesized using PrimeScript™ RT reagent Kit (TaKaRa, China). Quantitative real-time PCR was carried out using TB Green™ Premix Ex Taq™ II (TaKaRa, China) on ABI 7500 real-time PCR system (Applied Biosystems, USA). The relative expression was calculated using 2^−ΔΔCt^ method [61]. β-actin and U6 were used as reference genes.

### Western blotting

Total protein of cells or exosomes was isolated by using the cell lysis buffer (Beyotime Institute of Biotechnology, China). The western blotting assay was performed as previously reported [62]. The primary antibodies for IGF2BP3, CD63, and Tsg101 were obtained from Santa Cruz Biotechnology (Santa Cruz Biotechnology, USA). Optical density was quantified by the Uvitec Alliance software (Eppendorf, Germany).

### Cell transfection

Anti-sense oligonucleotide (ASO) for circFOXK2 knockdown and pcDNA3.1vectors for circFOXK2 overexpression were obtained from RiboBio (Guangzhou, China). The sequence information were as following: ASO-circFOXK2: 5′-GAAGGUGCACAUUCAGGUUTT-3′; ASO-negative control: 5′-TTCTCCGAACGTGTCACGT-3′; pcDNA3.1 vector-circFOXK2 (forward): 5′-GCGATATCGTGCACATTCAGGTTCCCGAG-3′, pcDNA3.1 vector-circFOXK2 (reverse): 5′-GCCCGCGGCTTCGGGCTGTCTCCA-3′. ASO negative control and pcDNA3.1vectors were used as the negative control, respectively. Lentiviral miR-370 mimic, inhibitor, respective negative control were obtained from Qiagen (Hilden, Germany). Plasmids for IGF2BP3 overexpression and siRNAs for IGF2BP3 knockdown were purchased from Qiagen (Hilden, Germany). Cell transfection was performed using Lipofectamine™ 3000 Transfection Reagent (Invitrogen, USA) according to the manufacturer’s instructions.

### Fluorescence *in situ* hybridization (FISH)

FISH assay was carried out as previously described [63]. In brief, circFOXK2-specific probes marked with Digoxigenin (DIG) -11-uridine triphosphate (UTP) (Roche, USA) was used. Cells were fixed with 4% paraformaldehyde for 10 min and permeabilized in 0.5% Triton X-100 in PBS solution for 5 min. Cells were then hybridized with the probe at 37°C for 16 hours. Then, the cells were washed with sodium citrate containing 0.1% Tween-20 for 5 min and then saline-sodium citrate (SSC) buffer for 5 min. Cells were stained with 4′,6-diamidino-2-phenylindole (DAPI) (Invitrogen, USA) for 10 min. Images were taken using SP8 laser confocal microscopy (Leica, Germany).

### Luciferase report assay

The conserved sequences containing the putative binding site of miR-370 were synthesized from the circFOXK2 sequence and then were cloned into pGL3-enhancer vector (Promega Corporation, USA). The mutated binding site of miR-370 was also cloned into the same luciferase reporter. Luciferase reporter plasmids and miR-370 mimic/negative control were transfected into HEK293T cell using Lipofectamine™ 3000 Transfection Reagent (Invitrogen, USA) according to the manufacturer’s instructions. After 24 hours of transfection, relative luciferase activity was determined using Dual-Luciferase^®^ Reporter Assay System (Promega Corporation, USA) according to the manufacturer’s instructions.

### RNA and circRNA pulldown

Biotin-labeled circFOXK2-specific probe and negative control probe were (Sangon Biotech, China) were used for RNA pulldown assay. The assay was performed as previously described [64, 65]. RNAs attached to the beads were isolated using Trizol (Invitrogen, USA) and measured by qRT-PCR. Proteins attached to the beads were measured by Western blotting.

### Exosome isolation and identification

Exosomes were isolated from BT-549 and BT-549-H cultured medium with exosome-free PBS using Total Exosome Isolation Reagent (Invitrogen, USA) according to the manufacturer’s instructions. Then, transmission electron microscopy (TEM) and nanoparticle tracking analysis (NTS) were performed to determine exosome morphology, and size, and concentration, as previously described [66]. The concentration of exosomal proteins was determined using Pierce BCA Protein Assay Kit (Thermo Fisher Scientific, USA).

### Exosome electroporation

MiR-370 mimic or inhibitor was loaded into exosomes through electroporation assay using Gene Pulser Xcell Electroporation Systems (BioRad, USA) as previously described [67].

### Cell-exosome coculture

BT-549 and BT-549-H cells (1 × 10^5^) were placed in the inner chamber of 24 transwell plates. Recipient cells were placed in the outer chamber. Twenty-four hours later, cells in the outer chamber were used to determine the migration and invasion abilities.

### Animal study

Male BALB/c athymic nude mice (6–8 weeks) were purchased from Shanghai Laboratory Animal Center (Shanghai, China) and maintained in standard conditions in the animal facility at our hospital. CircFOXK2-overexpressing/knockdown BT-549 or BT-549 cells (1 × 10^5^) were injected into mice through the lateral tail vein (*n* = 8 per group). Eight weeks post-injection, mice were sacrificed, and lung and liver tissues were collected. The number of tumor nodules was quantified through hematoxylin and eosin (HE) staining and immunohistochemistry (IHC) assay, as previously described [68, 69].

### Statistical analysis

Data were represented as mean ± SD. Data were analyzed by SPSS 18.0 software (SPSS Inc, USA). Pearson correlation was used to analyze the correlation between circFOXK2 and IGF2BP3, and circFOXK2 and miR-370. Mean differences between groups were analyzed using the Tukey’s test. In this study, differences were regarded to be significant at *p* < 0.05.
